# Do commonly used frailty models predict mortality, loss of autonomy and mental decline in older adults in northwestern Russia? A prospective cohort study

**DOI:** 10.1186/s12877-016-0276-4

**Published:** 2016-05-09

**Authors:** Anna Turusheva, Elena Frolova, Elena Korystina, Dmitry Zelenukha, Pulodjon Tadjibaev, Natalia Gurina, Eralda Turkeshi, Jean-Marie Degryse

**Affiliations:** Institut de Recherche Santé et Société, Université Catholique de Louvain, Clos Chapelle-aux-Champs, 30 bte 30.05, 1200 Woluwe-Saint-Lambert, Brussels Belgium; The North-Western State Medical University named after I.I. Mechnikov, St. Petersburg, Russia; Department of Public Health and Primary Care, KU Leuven, Leuven, Belgium

**Keywords:** Frailty, Mortality, Dependency, Disability, Russia

## Abstract

**Background:**

Frailty prevalence differs across countries depending on the models used to assess it that are based on various conceptual and operational definitions. This study aims to assess the clinical validity of three frailty models among community-dwelling older adults in north-western Russia where there is a higher incidence of cardiovascular disease and lower life expectancy than in European countries.

**Methods:**

The Crystal study is a population-based prospective cohort study in Kolpino, St. Petersburg, Russia. A random sample of the population living in the district was stratified into two age groups: 65–75 (*n* = 305) and 75+ (*n* = 306) and had a baseline comprehensive health assessment followed by a second one after 33.4 +/−3 months. The total observation time was 47 +/−14.6 months. Frailty was assessed according to the models of Fried, Puts and Steverink-Slaets. Its association with mortality at 5 years follow-up as well as dependency, mental and physical decline at around 2.5 years follow up was explored by multivariable and time-to-event analyses.

**Results:**

Mortality was predicted independently from age, sex and comorbidities only by the frail status of the Fried model in those over 75 years old [HR (95 % CI) = 2.50 (1.20–5.20)]. Mental decline was independently predicted only by pre-frail [OR (95 % CI) = 0.24 (0.10–0.55)] and frail [OR (95 % CI) = 0.196 (0.06–0.67)] status of Fried model in those 65–75 years old. The prediction of dependency and physical decline by pre-frail and frail status of any the three frailty models was not statistically significant in this cohort of older adults.

**Conclusions:**

None of the three frailty models was valid at predicting 5 years mortality and disability, mental and physical decline at 2.5 years in a cohort of older adults in north-west Russia. Frailty by the Fried model had only limited value for mortality in those 75 years old and mental decline in those 65–75 years old. Further research is needed to identify valid frailty markers for older adults in this population.

## Background

Following the aging population trend worldwide the number of older adults in Russia is increasing, and the percentage of retirement-age adults is expected to grow from 22.7 % of the population in 2014 to 28.7 % in 2031 [1]. Some people reach old age in good health and can remain independent and active participants in society, whereas others experience deterioration in their physical and cognitive functions that affects their ability to live independently [2]. This latter group of older adults requires additional assistance, leading to increased financial commitment for their treatment and maintenance. There is a need to develop tools that identify this group of frail older adults and implement strategies to help them [2–4].

Frailty is a state of decreased reserve and decline in multiple physiological systems that results in an increased risk of adverse outcomes such as falls, decreased mobility, slow recovery from any illness, reduced independence and increased hospitalization, disability, and death [3–10]. Frailty appears when an individual’s reserve capacity has decreased to a critically low point at which even small disturbances can result in a series of complications [4, 7]. Frailty, chronic disease and disability overlap, but are considered clinically distinct [7].

The prevalence of frailty differs across countries and depends on the diagnostic model used to assess it. More than 30 criteria have been proposed to identify or predict frailty in older adults [3]. These criteria have been included in a variety of frailty models based on various conceptual and operational definitions. Currently all frailty instruments can be divided into self-reported (including the Groningen Frailty Indicator (GFI) [9], the Tilburg Frailty Indicator [11] and the Sherbrooke Postal Questionnaire [12]) and performance-based ones [13]. There are two widespread performance-based instruments for measuring frailty in older adults: the phenotype frailty model and the frailty index of cumulative deficits [3]. The frailty phenotype model (the Fried model) is closely linked with sarcopenia and defines frailty as a biological syndrome of decreased reserve and resistance to stressors that results from cumulative declines across multiple physiological systems [7]. According to the phenotype model, frailty is not synonymous with either comorbidity or disability, but comorbidity is an etiological risk factor for frailty while disability is an outcome [5]. The cumulative deficit approach (e.g., the Puts [8] and Rockwood models [14]) was developed based on the concept of the number of health “deficits” that are manifested in an individual, leading to a continuous measure of frailty. Although not each health ‘deficit’ such as hearing impairment poses an obvious or imminent threat of mortality, they contribute cumulatively to an increased risk of functional decline and death.

There are only few population-based studies on the global health of older adults in Russia. Previous studies have addressed specific problems including the influence of social factors and lifestyle on the levels of anxiety and depression, sleep disorders and cognitive functions in people more than 60 years old [15–17]. However, these reports do not provide a comprehensive, reliable and clear picture of the different domains of the health status of older adults in Russia.

In the first ever cross-sectional study in Russia for this age group (the Crystal study) a higher burden of cardiovascular disease (86.7 %), depression (34.2 %) and different degrees of cognitive impairment (34.6 %) were found compared to European countries [18]. The prevalence of frailty in the Crystal study population was assessed through three different conceptual approaches to frailty: the frailty phenotype model (the Fried model) [7], the cumulative deficit approach (the Puts model) [8] and a self-assessment questionnaire (the 15-item Groningen Frailty Indicator (Steverink-Slaets model)) [9]. In the Crystal study population, the prevalence of frailty was found comparable to that identified in other international studies [18]. The prevalence of frailty was higher using the Puts model, whereas the prevalence of a pre-frail status was higher using the Fried model [18]. The aim of the current study is to assess the clinical validity of the three frailty models discussed above in terms of their association with mortality, dependency and mental decline among the community-dwelling older adults in the Crystal study population.

## Methods

### Study design and population

The Crystal study is the first prospective cohort study of community-dwelling individuals who are 65 and older living in the Kolpino district of St. Petersburg. The study began in January 2009 with the aim of providing a picture of the health status of community-dwelling older adults aged 65 and older in the St. Petersburg district identifying groups of people with immediate health needs who might benefit from geriatric prevention strategies and determining which of the frailty models is most applicable and informative in this Russian population.

**Fig. 1:**
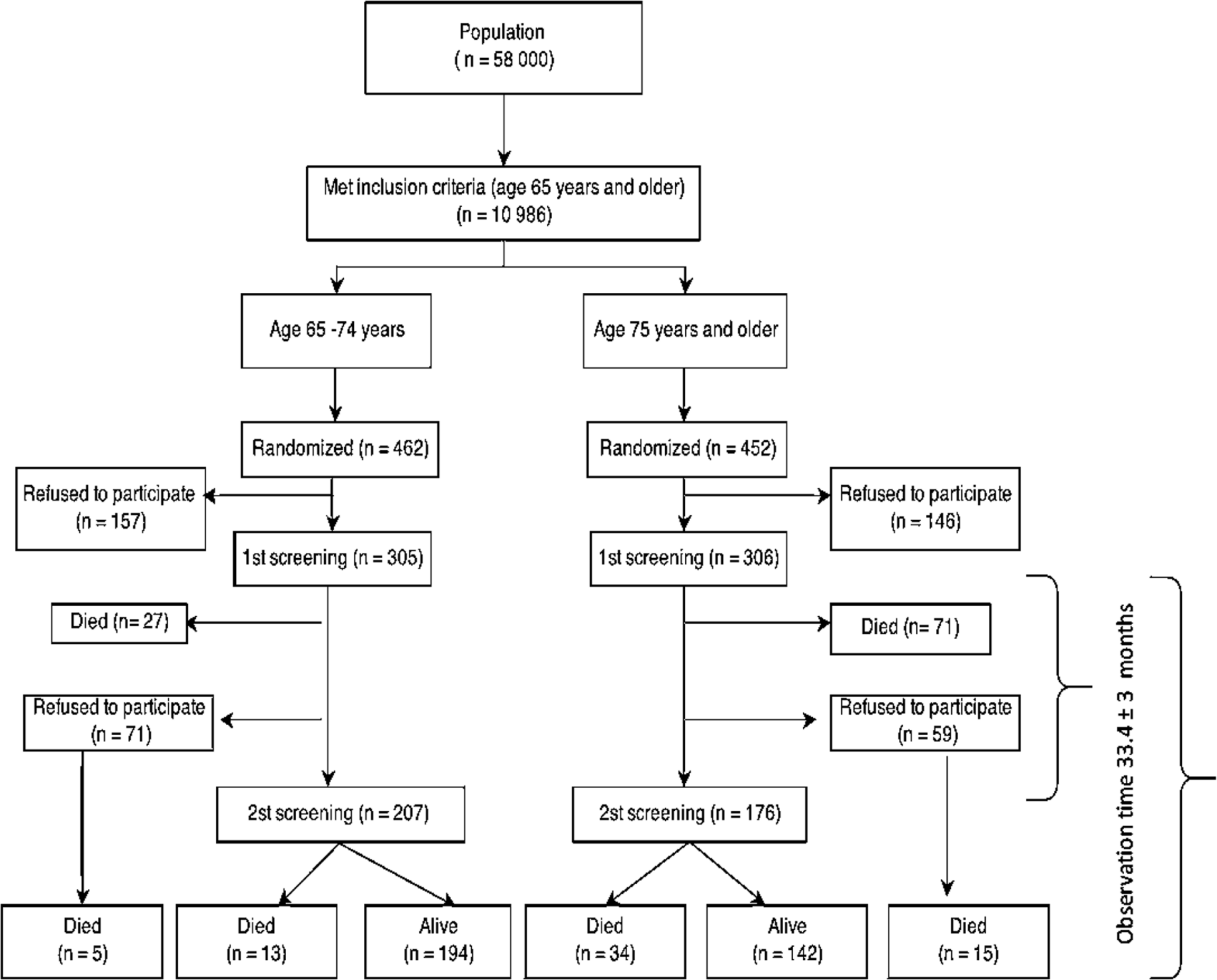
Flowchart of the data collection of the Crystal study

The primary care clinic (Policlinic no. 95) serves a population of 58,000 inhabitants based on a territorial concept of administration. Of that population 10,986 are aged 65 and older. As life expectancy in Russia is 64 years for men and 75 years for women largely due to the very high rate of cardiovascular mortality in working age people [1], the study population was stratified into two groups to compare those over 75 years old with the younger population (65–74 years). A representative random sample of 462 people in the younger group and 452 people in the older group was selected. No one was excluded based on health or cognitive function. The response rate was 66.2 % (*n* = 305) in the younger group (65–74) and 67.9 % (*n* = 306) in the older group (≥75). To test for sampling bias, those who agreed to participate in the study and those who were invited, but did not participate, were compared, and no significant difference was found in the sex and age distributions. Selected persons were invited to participate by telephone. Some people who were unable to come to the Policlinic were examined at home. Fourteen nurses were trained as clinical research assistants during 3 half-day training sessions to familiarize them with the questionnaires and test procedures. All data were collected from March to December 2009 (T0) (Fig. 1). A second assessment (T1) was performed an average of 33.4 ± 3 months after the date of the first data collection from February to August 2012. Out of the 611 participants included in the first assessment, 203 participants from the younger age group and 176 from the older age group were evaluable for the second assessment (102 participants died before the second assessment and 130 patients refused to participate) (Fig. 1). No difference was found between the baseline characteristics of participants who participate and who did not participate in the second assessment. The last update of mortality was in February 2014 (T2) and there was no loss of follow-up. The average total observation period of the study was 47 ± 14.6 months (T2). Other details of the sampling and data collection procedures have been already described [18]. The local ethics committee of The North-Western State Medical University named after I.I. Mechnikov approved this research for Postgraduate Studies and informed consent was obtained from all participants.

### Frailty models

Each participant in the Crystal study was assessed using the following models:

#### The frailty phenotype model (Fried model)

The assessment of frailty based on the Fried model [7] used the following five criteria:

- Weight loss was defined as unintentionally losing 6 kg of weight in the past 6 months or 3 kg in 3 months.
- Weakness defined as the lowest quintile for grip strength at baseline adjusted for sex and body mass index. In the Crystal study population the grip strength was measured with a carpal dynamometer (DK-50, Nizhni Tagil, Russian Federation) based on the standard protocol for the procedure presented in the Groningen Elderly Tests [19]. The maximum reading (kg) from three attempts for each hand was recorded separately, and the average of the left and right scores were calculated and analyzed.
- Poor endurance and energy was defined as a negative answer to the question 13 (“Do you feel full of energy?”) of the 15-item Geriatric Depression Scale (GDS-15) that was used to screen for depressive symptoms [20].
- Slowness was defined as being in the lowest quintile for walking speed adjusted for sex and height. Walking speed was tested as part of the Short Physical Performance Battery (SPPB) that included also the tests of rising from a chair, putting on and taking off a cardigan, and maintaining balance in a tandem stance. SPPB has been used and described in detail in several studies and is a reliable and valid measure of physical functioning [21]
- Low physical activity level was defined as self-reported low level of daily physical activity according to the question “How would the patient rate his/her own physical fitness?” in the Groningen Frailty Indicator questionnaire (GFI) [9]

Participants were considered frail if three or more of the above criteria were present pre-frail if one or two criteria were present and robust if they had none of the criteria [7].

#### The cumulative deficit approach (Puts model)

The frailty assessment according to the Puts model [8] was done using the following components:

- Low body weight was operationalized as a body mass index (BMI) <23. Standardized measurements of height and weight were performed for each Crystal study participant.
- Low peak expiratory flow was operationalized as the lowest sex-adjusted quintile of forced expiratory volume in 1 s (FEV1). Spirometry was done according to the American Thoracic Society/European Respiratory Society criteria [22] using two portable electronic spirometers (MIR Spirobank Rome, Italy). Four research nurses were trained to perform spirometry for all study participants.
- Poor cognition was defined as a mini mental status examination (MMSE) score of less than 24 [23]
- The presence of a vision problem a hearing problem or urinary incontinence based on the self-report of the participants
- Low mastery was defined as the lowest quintile of the Sense of Coherence scale (SOC) based on Antonovsky’s salutogenic theory which assesses how the way people view their lives influences their health [24].
- Depressive symptoms were assessed with the GDS-15 and a scores above five was considered suggestive of depression [20].
- Comorbidity was defined as its presence of two or more diseases. Details of past and current medical problems were collected based on anamnesis or information that was present in the medical records. Information on angina pectoris myocardial infarction, arrhythmias, peripheral artery disease, stroke, obstructive pulmonary disease or asthma, diabetes mellitus, cancer, osteoarthritis and rheumatoid arthritis was systematically documented.

The participants with one or two criteria were considered pre-frail those with three or more were considered frail and those with none were classified as robust [8].

#### The “index” approach: (Steverink-Slaets model)

The Steverink-Slaets model in the format of the Groningen Frailty Indicator (GFI is a self-reported questionnaire that estimates eight domains: mobility, physical fitness, vision, hearing, nourishment, morbidity, cognition, and psychosocial [9]. The 15 items are scored 1 (absence) or 0 (presence) of a problem in a particular domain. The model was developed as a screening instrument (frailty index). A score greater than 5 identifies that a person is frail, a score of less than 4 as robust, and a score of 4 or 5 as pre-frail.

### Outcome measures

Mortality data were obtained from the official reports of Policlinic no. 95 in Kolpino St.Petersburg.

#### Decline of independence in activities of daily living

The Barthel Index of the activities of daily living was used to determine participants’ baseline level of functioning and as a consequence, their degree of dependence [25]. The cutoff for dependence was defined as a score of less than 95 [26]. A significant decline in independence was defined as incident dependence that appeared between T0 and T1.

#### Mental decline

Mental decline was defined as a decline in either the MMSE score or the GDS-15 test score. A relevant decline in MMSE was determined using the Edwards-Nunnally index [27]. This index determines the probability of substantial individual change and avoids the problem of regression to the mean. Based on the scale reliability and the 95 % CI of the mean score at T0 the index computes whether a significant change between T0 and T1 has occurred. The cut-off for the GDS-15 was defined as a score of more than 5 [20]. Subjects who shifted from GDS-15 < 5 at baseline to GDS-15 > 5 at T1 were defined as having a significant worsening in depression status.

#### Physical decline

Physical decline was defined as a significant change between T0 and T1 in either the SPBB score or in the average grip strength of both hands. Significant declines in muscle strength and SPPB scores were determined using the Edwards-Nunnally index [27].

#### Covariates

##### Multimorbidity

Details of past and current medical problems were collected based on anamnesis or information presented in the medical records. Information on coronary artery diseases myocardial infarction, arterial fibrillation, peripheral artery disease, stroke, obstructive pulmonary disease or asthma, diabetes mellitus, cancer, Parkinson, osteoarthritis and rheumatoid arthritis was systematically documented. A disease count was used as an index of multimorbidity A three- point ordinal scale was used based on the distribution of the disease count (DC): Level 1: DC <3; level 2: DC 3–4; level 3: DC >5 [28].

### Statistical analysis

A rigorous procedure of “data -cleaning” was used. The test-retest reliability of the different measurements was taken into account in our analysis. Participants were categorized in three groups (frail pre-frail, robust) according to each of the three frailty models. The agreement between the three frailty models was estimated using Cohen’s Kappa statistic and was considered as excellent for Kappa values of 0.81–1; good for 0.61–0.80; moderate for 0.41–0.60; slight for 0.21–0.40; and poor for values lower than 0.21 [29]. Kaplan-Meier curves were used to visualize survival for the three groups according to each model for both age groups. Significance of differences was evaluated with the log-rank test. Cox proportional hazard models were used to investigate the association between frail and pre-frail status at baseline and mortality after adjusting for sex, age and all individual morbidities. The robust group was used as a reference category. The Schoenfeld residuals and log-log plot of survival were used to test the proportional hazards assumption. Differences between the groups of participants were compared using Student’s t test (for continuous variables) or chi-squared tests (for categorical variables). The relationship between frailty and a physical and mental decline was examined using logistic regression analysis.

In order to investigate a possible bias due to selective mortality of frailer people between the assessments at t0 and t1 we performed a sensitivity analyses using a “best” and “worst” case scenario. We performed the logistic regression again under the assumption that (1) all of the persons that died between the first and the second assessment remained stable (best case) and (2) that all of the persons that died between the first and the second assessment declined (worst case). Statistical calculations were performed using SPSS 20.0 (SPSS Inc., Chicago, IL, USA) and MedCalc 11.5.00 (Medcalc Software, Oostende) and *p*-values < 0.05 were considered significant.

## Results

### Baseline characteristics of the study population

A summary of the health status of the study participants is presented in the Table 1. Briefly at the baseline 611 participants in total were included in the Crystal study. The number of females was 2.5 times higher than the number of males in the both age group. The median age was 70 [68–72] years in the younger age group and 79 [77–83] years in the older age group. We have observed that in the younger age group the prevalence of stroke, cognition impairment, anemia, vision and hearing problem, incontinence and person in risk of malnutrition was higher than in the older age group. On the other hand, the persons from the older age group had a higher prevalence of arterial fibrillation, diabetes, depression and the score of the Barthel index less than 95.

**Table 1:** Health characteristics of older adults in the Crystal study, according to the age group

	65–74 years old (*n* = 305)	75 years and older (*n* = 306)
Sex		
Male, *n* (%)	100 (32.8)	73 (23.9)
Age (years), median, [IQR]	70 [68–72]	79 [77–83]
BMI (kg/m^2^) mean, ±SD	29.1 ± 4.9	28.1 ± 4.9
Comorbidities		
Coronary artery disease, *n* (%)	228 (74.8)	256 (83.7)
Myocardial infarction, *n* (%)	37 (12.1)	40 (13.1)
Atrial fibrillation, *n* (%)	97 (31.8)	87 (28.4)
Stroke, *n* (%)	37 (12.1)	54 (17.6)
Diabetes mellitus, *n* (%)	49 (16.1)	38 (12.4)
COPD, *n* (%)	71 (21.3)	77 (23.2)
Asthma, n (%)	10 (3.3)	17 (5.6)
Peripheral arterial disease, *n* (%)	69 (22.6)	67 (21.9)
Osteoarthritis or arthritis (%)	7 (2.3)	17 (5.6)
Cancer, *n* (%)	11 (3.6)	11 (3.6)
Parkinson’s, *n* (%)	2 (0.7)	5 (1.6)
Renal pathology, *n* (%)	21 (6.9)	10 (3.3)
Vision impairment, *n* (%)	99 (32.5)	128 (41.8)
Hearing impairment, *n* (%)	61 (20.1)	101 (33.0)
Incontinence, *n* (%)	104 (34.1)	146 (47.7)
SPPB score, median, [IQR]	10 [7–12]	7 [5–10]
Grip strength, median, [IQR]		
Males	29.2 [24.1–33.1]	22.5 [16.9–28.4]
Females	16.0 [12.3–19.3]	12.3 [9.3–16.3]
Barthel Index < 95, *n* (%)	44 (14.4)	101 (33.0)
GDS-15 score > 5, *n* (%)	79 (25.1)	130 (42.5)
MMSE score, *n* (%)		
0–9	1 (0.3)	7 (2.3)
10–20	20 (6.6)	58 (19.0)
21–24	40 (13.1)	85 (27.8)
25–30	244 (80)	156 (51.0)
SOC score, mean ± SD	63.9 ± 9.7	64.2 ± 11.6
FEV1, L, mean ± SD	2.2 ± 0.7	1.8 ± 0.6
Frailty status according to Puts model		
Robust, *n* (%)	65 (22.0)	17 (5.7)
Pre–frail, *n* (%)	117 (39.5)	98 (33.1)
Frail, *n* (%)	114 (38.5)	181 (61.1)
Frailty status according to Fried model		
Robust, *n* (%)	55 (18.3)	36 (12.2)
Pre–frail, *n* (%)	196 (65.1)	175 (59.1)
Frail, *n* (%)	50 (16.6)	85 (28.7)
Frailty status according to Steverink-Slaets model		
Robust, *n* (%)	168 (55.4)	90 (29.4)
Pre–frail, *n* (%)	62 (20.5)	88 (29.0)
Frail, *n* (%)	73 (24.1)	125 (40.8)

*BMI* body mass index, *GDS15* geriatric depression scale 15 items, *MMSE* mini mental status examination, *COPD* chronic obstructive pulmonary disease, *FEV1* forced expiratory volume in 1 s, *SOC* sense of coherence scale

The agreement between the different indexes for the detection of a frail and pre - frail status was poor with Kappa coefficients ranging from 0.14 (95 % CI: 0.06–0.21) for the Fried and the Puts models to 0.30 (95 % CI: 0.23–0.37) between the Steverink-Slaets and Puts models in the younger age group. Kappa coefficients in the older age group were ranging from 0.16 (95 % CI: 0.87–0.24) for the Fried and the Puts models to 0.32 (95 % CI: 0.25–0.40) between the Steverink-Slaets and Puts models.

#### Mortality

**Fig. 2:**
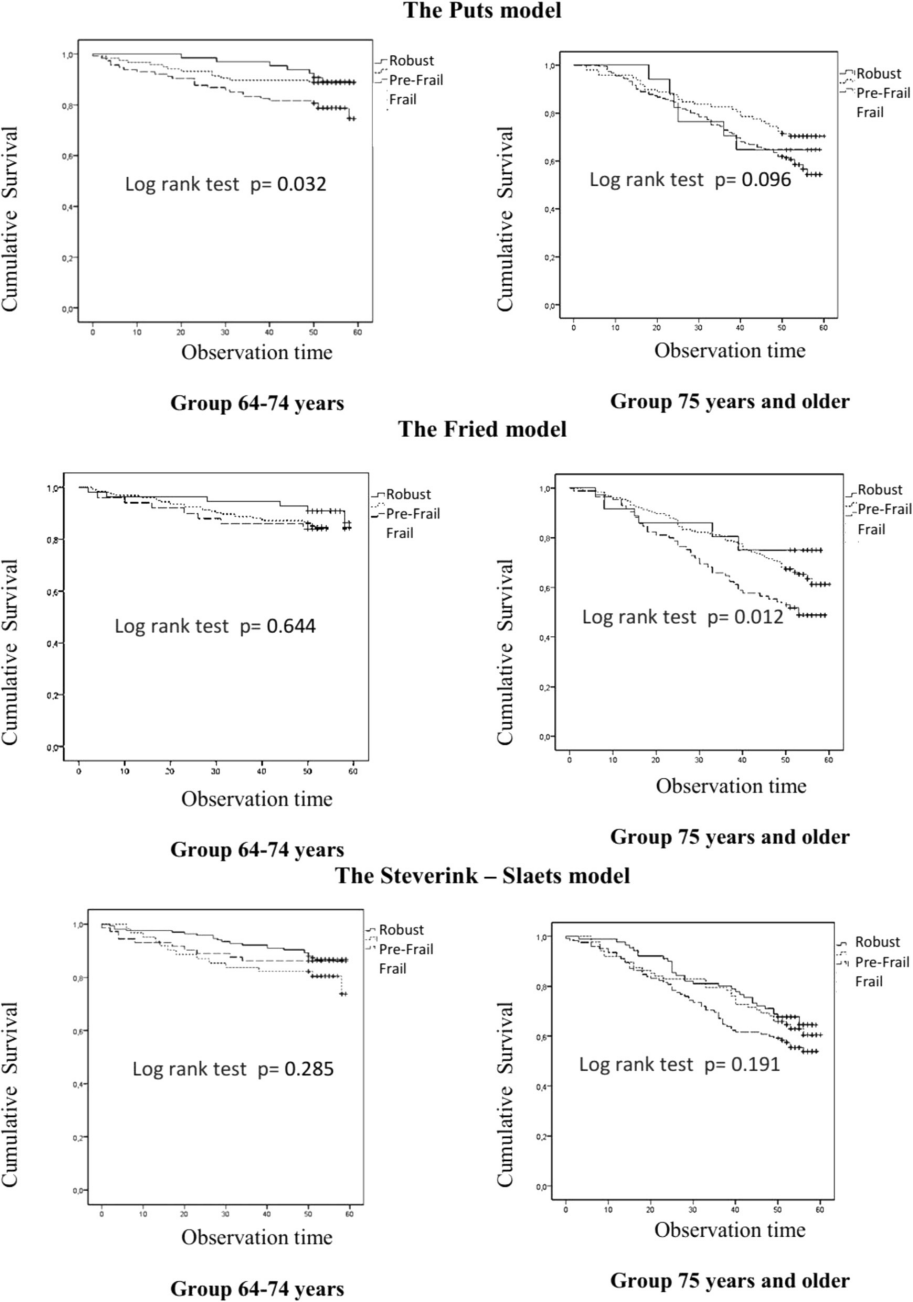
Kaplan – Meier survival curves comparing overall survival of frail, pre-frail and robust persons according to the Puts, Frieds and Steverink–Slaets models in the two age groups

Data about mortality were available for all participants. During the follow-up period 165 (27 %) patients died. In the older group, we found that only the Fried model predicted the mortality of frail participants even after adjusting for age, sex and comorbidities [HR (95 % CI) = 2.50 (1.20–5.20); *p* = 0.014] (Fig. 2, Table 2). There was an association between the Puts model and a greater risk of death among frail participants in the younger age group (Fig. 2), but this difference disappeared after adjusting for sex and age. The Steverink-Slaets model did not predict mortality in either age group (Fig. 2).

**Table 2:** Cox proportional hazard model analysis for the association between frailty according to Frieds models and mortality in those 75 years and older

	Model 1	Model 2
	HR (95 % CI)	HR (95 % CI)
Pre-frail	1.61 (0.80–3.24)	1.64 (0.81–3.31)
Frail	2.39 (1.11–4.93)*	2.50 (1.20–5.20)*
Sex	2.17 (1.44–3.25)**	2.13 (1.42–3.20)**
Age	1.11 (1.06–1.17)**	1.11 (1.06–1.16)**
Multimorbidity		0.94 (0.81–1.09)

1 – Model 1: Unadjusted + age, sex

2 – Model 2: Model 1 + number of comorbidities at the individual level

*HR* hazard ratios, *CI* confidence interval

*p < 0.05; **p < 0.001

**Fig. 3:**
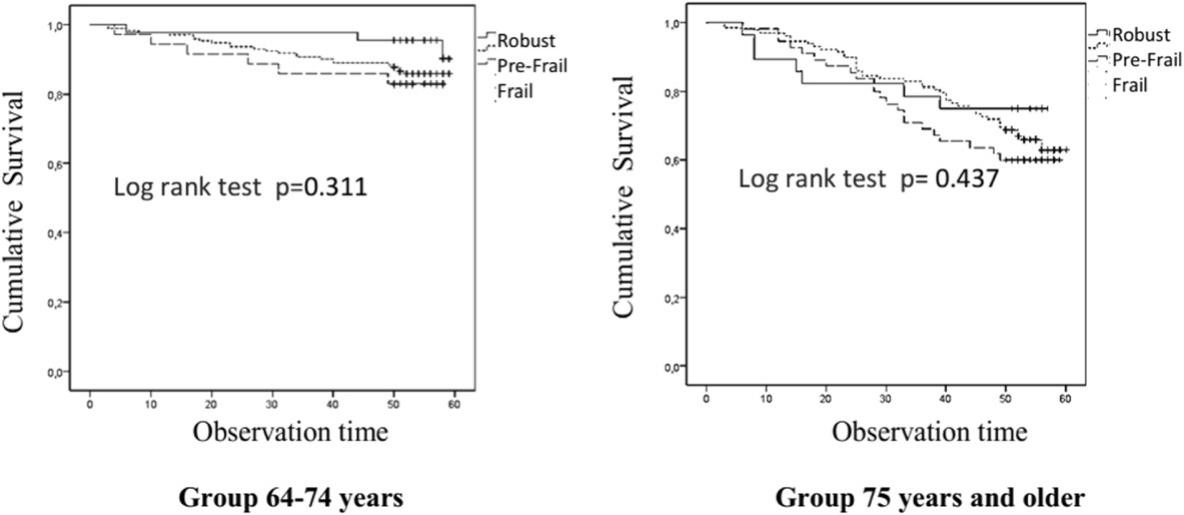
Kaplan – Meier survival curves comparing overall survival in frail, pre-frail and robust persons according to the Fried model after excluding participants with stroke, MMSE < 18, or a history of Parkinson’s disease or cancer

After excluding participants with stroke an MMSE score < 18 or a history of Parkinson’s disease or cancer (53 participants in the younger age group and 87 in the older age group), following the original Fried study [4], the association with mortality was not longer statistically significant in either age group (Fig. 3). Those with stroke, MMSE score < 18, or a history of Parkinson’s disease or cancer were more likely to show signs of malnutrition or risk of malnutrition, depression, higher levels of multimorbidity, prevalence of different frailty components of the Fried model and frailty based on other models (Table 3).

**Table 3:** Health characteristics of subjects with and without stroke, MMSE < 18, or a history of Parkinson’s disease or cancer

Characteristic	Without *n* = 471 (77.1)	With *n* = 140 (22.9)
Demographic		
Sex		
Male, *n* (%)	127 (27.0)	97 (69.3)
Age (years), mean ± SD	77.3 ± 5.8	79.4 ± 6.3
Mortality**	109 (23.1)	56 (40.0)
Medical problems		
*Cardiovascular diseases, n (%):*		
Coronary artery disease	371 (78.8)	113 (80.7)
Myocardial infarction	52 (11.0)	25 (17.9)
Arterial fibrillation	147 (31.2)	37 (26.4)
Multimorbidity, n (%)**:		
0–3	418 (88.7)	108 (77.1)
4–5	51 (10.8)	29 (20.7)
6–10	2 (0.4)	3 (2.1)
BMI (kg/m2), mean ± SD	28.9 ± 5.0	27.4 ± 4.5
MNA**		
< 17 “Malnourished”	4 (0.8)	7 (5.0)
17–23.5 “At risk of malnutrition”	79 (16.8)	27 (19.3)
> 23.5 “Normal nutritional status”	388 (82.4)	106 (75.7)
GDS-15 score >5, *n* (%)**	142 (30.1)	67 (47.9)
SPBB score, mean ± SD	8 ± 3.2	7 ± 3.6
Anemia, *n* (%)	90 (19.1)	27 (19.3)
FEV1 < LLN, *n* (%)	70 (14.9)	19 (13.6)
eGFR (MDRD) <60 mL/min/1.73 m^2^, *n* (%)	82 (17.4)	33 (23.6)
*Components of the Fried model*		
Weight loss, *n* (%)	61 (13.0)	24 (17.1)
Weakness, *n* (%)**	81 (17.2)	41 (29.3)
Poor endurance and energy, *n* (%)**	288 (61.1)	99 (70.7)
Slowness, *n* (%)	115 (24.4)	32 (22.9)
Low physical activity level, *n* (%)	191 (40.6)	83 (59.3)
Frailty categories according to Fried model, *n* (%)**		
Robust	72 (15.6)	19 (14.0)
Pre-frail	299 (64.9)	72 (52.9)
Frail	90 (19.5)	45 (33.1)
*Components of Puts model (Index)*		
Low body weight, *n* (%)	59 (12.6)	23 (16.5)
Low peak expiratory flow, *n* (%)	86 (18.5)	31 (22.3)
Poor cognition, *n* (%)	134 (28.5)	77 (55.0)
Vision problem, *n* (%)	170 (36.1)	57 (40.7)
Hearing problem, *n* (%)	125 (26.5)	37 (26.4)
Incontinence, *n* (%)	190 (40.3)	60 (42.9)
Low mastery, *n* (%)	99 (21.3)	32 (23.9)
Depressive symptoms, *n* (%)**	200 (42.5)	82 (58.6)
Comorbidity, *n* (%)**	135 (28.7)	71 (50.7)
Frailty categories according to Puts model, *n* (%)**		
Robust	74 (16.1)	8 (6.0)
Pre–frail	175 (38.1)	40 (30.1)
Frail	210 (45.8)	85 (63.9)
Categories of frailty according to Steverink-Slaets model, *n* (%)**		
Robust	213 (45.6)	45 (32,4)
Pre–frail	116 (24.8)	34 (24.5)
Frail	138 (29.6)	60 (43.2)

Differences between the groups of participants were compared using Student’s t test (for continuous variables) or chi-squared tests (for categorical variables)

*BMI* body mass index, *MNA* mini nutritional assessment, *GDS-15* geriatric depression scale 15 items, *SPPB* short physical performance battery, *FEV1* forced expiratory volume in 1 s, *LLN* lower limit of normal, *MDRD* The Modification of Diet in Renal Disease formula

**p < 0.05

Pre-frail status according to the three models did not predict mortality (Fig. 2).

#### Mental decline

Mental decline at 33.4 ± 3 months follow-up was predicted by pre-frail (OR (95 % CI) = 0.24 (0.10–0.55); *p* = 0.001) and frail (OR (95 % CI) = 0.196 (0.06–0.67); *p* = 0.009) status as defined by the Fried model even after adjusting for age sex and comorbidity only in the younger age group. The association with mental decline remained significant for pre-frail [OR (95 %CI) = 0.21 (0.08–0.52); *p* = 0.001] and frail [OR (95 %CI) = 0.13 (0.03–0.62); *p* = 0.010] participants even after applying the exclusion criteria of Fried [4].

#### Autonomy and physical decline

After 2.5 years of observation 3.9 % of participants from the younger age group and 13.1 % from the older age group developed dependency in performance of activities of their daily living and almost 50 % had a physical decline in the both age group. Pre-frail and frail status according to the three frailty models did not predict dependency or physical decline.

Our findings concerning mental physical and functional decline appear to be robust since in the best and worst case scenarios of our sensitivity analysis none of the frailty models predict mental, physical decline or loss of autonomy (Appendix).

## Discussion

### Main findings

In our cohort of community-dwelling older adults in the northwestern region of Russia we found a significant association between frailty and mortality only when using the Fried model for those ≥ 75 years old. Nonetheless, after excluding participants with stroke, a MMSE score < 18, and history of Parkinson’s disease or cancer, this association was not longer statistically significant. Pre–frail state according to any of the three models was not associated with mortality in either age group. Only frail and pre-frail states as defined by the Fried model predicted mental decline in the younger group. None of the three frailty models predicted new incidents of dependency, mental or physical decline during the 3 years of follow-up.

### Interpretation of findings in relation to previously published studies

In our study we analyzed three different approaches to defining frailty. The Fried model is a classical phenotype model that estimates only the physical status of older people, the Puts model is an accumulation deficit model, while he Steverink-Slaets model is an approach to measuring frailty in the primary care setting that uses a self-assessment questionnaire. All three models have previously been found to have an association with mortality in adults ≥ 65 years old [7–9, 30, 31]. In a recent systematic literature review, a higher mortality risk was reported for frail participants based on the phenotype model versus the accumulation deficit model [31]. Nevertheless, the predictive value of both types of frailty definitions was approximately 70 % in receiver operating characteristic curve areas [31, 32]. The specificity and sensitivity of GFI has also been previously estimated showing low positive and negative predictive values for identifying frail persons [23, 32, 33, 34]. We found a poor agreement between the three frailty models. This finding is consistent with results of the SAFEs cohort study where the agreement between Winograd’s, Donini’s, Rockwood’s and Schoevaerdts’s indices for the detection of frailty was also very poor, with Kappa coefficients ranging from −0.02 to 0.15 [29].

One recent study showed that the presence of cognitive impairment increases the likelihood of adverse outcomes in older patients [31, 35]. We found that the frail and pre-frail statuses based on the Fried model predicted mental decline in the younger group, although this model estimates only the physical status of older adults, which differs from the other models that we used in our study. This result is in line with findings from other studies that reported an association between low physical activity and cognitive impairment in older adults [36–39]. Clouston et al. reported that decreased grip strength was more strongly correlated with changes in the MMSE score [36].

The finding that the Fried model was not associated with mental decline in the older group may be explained by the development of dementia at a younger age and a higher prevalence of cognitive impairment in this population before reaching 75 years old. According to our findings the prevalence of cognitive impairment in the older group was significantly higher (49 %) than in the younger group (20 %) (Table 1).

Physical exercise and fitness have been proposed as potential factors that promote healthy cognitive aging [38, 39].

### Reasons why these frailty models lose their validity in Russia

The reasons why none of the frailty models employed in our study were clinically valid may be linked to the medical social and demographic profiles of Russians and differences between Russia and other countries.

In the past 20 years in most countries, overall life expectancy has increased, whereas in Russia, it has decreased by 3.6 years for men and 1.1 years for women between 1990 and 2006 [36]. Russia follows the worldwide trend of population aging, but in contrast to Europe, reductions in the total population and high mortality rates of the working population are the main contributors to this demographic shift [40–42]. In 2009, the mortality rate of the working population in Russia was 3 times higher than that of other European countries [43, 44].

Cardiovascular disease (CVD) is the main cause of death among those under 65 years old in Europe with 31 % of deaths before the age of 65 in men and 26 % in women [43]. The same trend is found in the Russian population but the CVD mortality rate is even higher, with 38.8 % of males and 36.8 % of females dying from CVD in the same age group [44]. In St. Petersburg, in 2009, the CVD mortality among men in this age group was 47.0 %, which is significantly higher than the average in Russia [44].

Thus Russia combines features of developed and developing countries, with a high mortality rate from non-communicable diseases, which resembles developed countries, and a low overall expectancy life, as in developing countries [40, 42].

A number of studies have reported associations between socio-economic status and health in adulthood and old age showing consistent evidence that the socio-economically disadvantaged have more chronic diseases, higher mortality rates and higher levels of frailty [45]. The influence of unfavorable socio-economic conditions and education level on health and mortality among the Russian population was shown in several studies [46, 47].

Therefore the participants in our study are people over 65 years old who survived the critical period of high burden of CVD diseases and the lack of necessary medical and surgical treatment [48] in the context of serious socio-economic stress and uncertainty concerning the future.

### Screening for frailty

Routine identification of frailty is recommended by international guidelines [49]. However the lack of consensus regarding the definition of frailty results in clinical and research challenges [6]. The frailty phenotype and index are basic ways of identifying frail persons [50]. However, it is inappropriate to consider the frailty phenotype and index as alternatives and/or substitutes for one another as each has pros and cons. The frailty phenotype may serve for the initial risk stratification of the population to different profiles (i.e., robust, pre-frail and frail), but it does not provide any indication regarding preventive or therapeutic interventions. Although the assessment of the frailty phenotype consists of simple tasks, its administration and meaningfulness may sometimes be problematic. The evaluation of muscle strength and gait speed is not always possible, particularly in primary care, due to the lack of dynamometers and/or space/time to use them [50]. The frailty index may serve as a more useful tool for ascertaining the effectiveness of interventions and describing health status trajectories over time, but it can be administered only after a comprehensive clinical assessment. Thus, these two frailty instruments should be considered as complementary [49].

Comprehensive geriatric assessment refers to a multidimensional and typically interdisciplinary, diagnostic process designed to assess the medical conditions, mental health, functional capacity and social circumstances of older adults [50]. As it encompasses both the frailty phenotype and index approaches – including different aspects of health status such as mental and social status – and as it aims to gather information in order to design appropriate interventions and support, it may be a more appropriate approach to identify older persons at risk for functional decline.

### Strengths and limitations

Our study has certain limitations. We did not have information on exact causes of death. The short period of time between first and second screening (33.4 ± 3 months) may have influenced the lack of association between different frailty models and incidents of dependency as well as mental and physical decline. “The low level of activity” in the Fried model was measured using the self reported low level of physical activity according to the answer to the correspondingquestion in the Groningen Frailty Indicator Questionnaire (GFI) instead of the Minnesota Leisure Time Activities Questionnaire as in the original article of L. Fried with colleagues. This may lead to some misclassification and biased results of our study.

The strengths of our study are its prospective design, the comprehensive assessment performed, the follow-up regarding mortality data up to 5 years and no loss of participant mortality data.

## Conclusion

In this first prospective cohort study of community-dwelling older adults in the northwestern region of Russia we have not been able to confirm that the frail and pre-frail status according to the Fried, Puts and Steverink-Slaets models of frailty are good predictors of mortality, dependency, as well as physical and mental decline. More research is required to understand the characteristics of frail older adults in Russia and identify the frailty markers for this population.

## Availability of data and materials

The privacy and data protection legislation in the Russian Federation does not allow us to publish the dataset in the public domain (Presidential decree of the Russian Federation from April 20, 2014 of No. 259/Letter of Ministry of Health 15 July N 21-1/10/2-3431). The dataset supporting the conclusions of this article are available upon request for replication purposes after signature of a formal agreement.

## Appendix Results of the sensitivity analysis concerning the associations between frailty and mental, physical or functional decline

**Table 4:** The worst-case scenarios of physical, mental and autonomy decline in frail, pre-frail and robust persons according to the Puts, Fried and Steverink–Slaets models in the Crystal population

	Mental decline OR (95 % CI)	Autonomy decline OR (95 % CI)	Physical decline OR (95 % CI)
Puts model			
Frail	1.68 (0.94–3.01); *p* = 0.08	0.73 (0.47–1.14); *p* = 0.16	1.47 (0.84–2.57); *p* = 0.18
Pre - frail	1.44 (0.79–2.64); *p* = 0.24	0.34 (0.16–0.71); *p* = 0.05	1.23 (0.69–2.21); *p* = 0.47
		(0.38 (0.24–1.21); *p* = 0.13)*	
The Steverink – Slaets model			
Frail	1.22 (0.79–1.86); *p* = 0.36	2.08 (1.92–3.34); *p* = 0.003	1.08 (0.71–1.64); *p* = 0.73
		(1.54 (0.91–2.60); *p* = 0.11)*	
Pre - frail	1.40 (0.89–2.22); *p* = 0.15	1.93 (1.55–3.21) *p* = 0.012	1.49 (0.94–2.35); *p* = 0.09
		(1.38 (0.80–2.40); *p* = 0.13)*	
Fried model			
Frail		1.37 (0.72–2.60); *p* = 0.34	
Pre - frail		2.44 (1.21–4.94); *p* = 0.013	
		(1,73 (0.82–3.64); *p* = 0.15)*	

*After adjustment for age and sex

**Table 5:** The best-case scenarios of physical, mental and autonomy decline in frail, pre-frail and robust persons according to the Puts, Frieds and Steverink–Slaets models in the Crystal population

	Mental decline OR (95 % CI)	Autonomy decline OR (95 % CI)	Physical decline OR (95 % CI)
Puts model			
Frail	0.89 (0.45–1.75); *p* = 0.74	2.27 (0.51–10.12); *p* = 0.28	0.81 (0.45–1.48); *p* = 0.50
Pre - frail	1.09 (0.54–2.17); *p* = 0.81	2.62 (0.57–11.94); *p* = 0.21	0.93 (0.50–1.72); *p* = 0.81
The Steverink – Slaets model			
Frail	0.62 (0.37–1.06); *p* = 0.82	1.98 (0.79–4.96); *p* = 0.15	0.59 (0.37–0.96); *p* = 0.03
			(0.60 (0.36–0.99); *p* = 0.44)*
Pre - frail	0.96 (0.56–1.63); *p* = 0.87	2.41 (0.94–6.17); *p* = 0.67	1.04 (0.643–1.68); *p* = 0.89
Fried model			
Frail		0.96 (0.26–3.55); *p* = 0.96	
Pre - frail		1.19 (0.39–3.59); *p* = 0.76	

*After adjustment for age and sex

## Abbreviations

BMI: body mass index
CI: confidence interval
CVD: cardiovascular disease
FEV1: forced expiratory volume in 1 s
GDS 15: the 15-item Geriatric Depression Scale
GFI: the Groningen Frailty Indicator
HR: hazard ratio
LLN: lower limit of normal
MDRD: The Modification of Diet in Renal Disease formula
MMSE: the Mini-Mental State Examination
MNA: the Mini Nutritional Assessment
OR: odds ratio
SD: standard deviation
SOC: the Sense of coherence scale
SPPB: the Short Physical Performance Battery
T0: date of the first screening
T1: date of the second screening
T2: the last update of mortality data
